# Kilohertz serial crystallography with the JUNGFRAU detector at a fourth-generation synchrotron source

**DOI:** 10.1107/S2052252523008618

**Published:** 2023-10-14

**Authors:** Filip Leonarski, Jie Nan, Zdenek Matej, Quentin Bertrand, Antonia Furrer, Ishkhan Gorgisyan, Monika Bjelčić, Michal Kepa, Hannah Glover, Viktoria Hinger, Thomas Eriksson, Aleksander Cehovin, Mikel Eguiraun, Piero Gasparotto, Aldo Mozzanica, Tobias Weinert, Ana Gonzalez, Jörg Standfuss, Meitian Wang, Thomas Ursby, Florian Dworkowski

**Affiliations:** a Photon Science Division, Paul Scherrer Institut, CH-5303 Villigen PSI, Switzerland; b MAX IV Laboratory, Lund University, POB. 118, SE-22100 Lund, Sweden; c Division of Biology and Chemistry, Paul Scherrer Institut, CH-5303 Villigen PSI, Switzerland; d Scientific Computing, Theory and Data, Paul Scherrer Institut, CH-5303 Villigen PSI, Switzerland; SPring-8, Japan

**Keywords:** macromolecular crystallography, time-resolved serial crystallography, X-ray detectors, fourth-generation synchrotrons, JUNGFRAU detector, protein structure, data networks

## Abstract

Serial and time-resolved macromolecular crystallography are on the rise. However, beam time at X-ray free-electron lasers is limited and most third-generation synchrotron-based macromolecular crystallography beamlines do not offer the necessary infrastructure yet. Here, a new setup is demonstrated, based on the JUNGFRAU detector and Jungfraujoch data-acquisition system, that enables collection of kilohertz serial crystallography data at fourth-generation synchrotrons. More importantly, it is shown that this setup is capable of collecting multiple-time-point time-resolved protein dynamics at kilohertz rates, allowing the probing of microsecond to second dynamics at synchrotrons in a fraction of the time needed previously. A high-quality complete X-ray dataset was obtained within 1 min from lysozyme microcrystals, and the dynamics of the light-driven sodium-pump membrane protein KR2 with a time resolution of 1 ms could be demonstrated. To make the setup more accessible for researchers, downstream data handling and analysis will be automated to allow on-the-fly spot finding and indexing, as well as data processing.

## Abstract 2

The first demonstration of 2 kHz time-resolved serial crystallography data acquisition at a fourth-generation synchrotron, using the JUNGFRAU 4M pixel detector.

## 1. Introduction

The development of X-ray free-electron lasers (XFELs) has initiated a renaissance of time-resolved macromolecular crystallography (MX) experiments at physiological conditions (Chapman *et al.*, 2011 ▸; Boutet *et al.*, 2012 ▸; Orville, 2020 ▸; Pearson & Mehrabi, 2020 ▸; Barends *et al.*, 2022 ▸). While their ultra-short intense burst of X-rays pushed the time resolution to the femtosecond domain (Milne *et al.*, 2017 ▸), it also created new challenges in terms of sample delivery and consumption, since thousands of crystals need to be delivered to the XFEL pulse, sparking many novel ideas for serial sample delivery to the X-ray beam. These sample-delivery systems include the high-viscosity extruder (HVE) (Weierstall *et al.*, 2014 ▸), fixed target chips (Martiel *et al.*, 2019 ▸) and tape drives (Beyerlein *et al.*, 2017 ▸). Another major bottleneck of the technique is the sparse availability of beam time at XFEL facilities. To mitigate these challenges, serial synchrotron crystallography (SSX) was actively developed at many synchrotron facilities to make use of the novel sample-delivery systems and experiment tech­niques, while offering significantly fewer hurdles for users to access the technique (Weinert *et al.*, 2017 ▸; Botha *et al.*, 2015 ▸). However, time-resolved experiments at synchrotron sources are essentially limited by the photon flux, radiation damage and the detector readout rate. The Paul Scherrer Institut (PSI) has been at the forefront of state-of-the-art hybrid pixel-array detector development for nearly two decades now. The PILATUS and EIGER photon-counting detectors have revolutionized diffraction data collection at synchrotrons worldwide, and the JUNGFRAU integrating pixel detector (Mozzanica *et al.*, 2018 ▸) has applied the same key technologies in the field of XFELs. MAX IV, on the other hand, is the pioneer in the next generation of synchrotron light sources, providing the most brilliant beams in a micrometre focus. Bringing the two competencies together is a great opportunity to bridge the gap between XFELs and third-generation synchrotrons, allowing a new chapter of structural biology, including ultra-high-throughput screening, serial crystallography structure determination of true microcrystals, and especially time-resolved MX, even in the microsecond regime.

### 1.1. Fourth-generation synchrotrons

The MAX IV 3 GeV storage ring (Tavares *et al.*, 2014 ▸) was the first of the new fourth-generation synchrotron light sources based on multi-bend achromat designs (Borland *et al.*, 2014 ▸) when it went into operation in 2016. These diffraction-limited sources are characterized by a low emittance, high brilliance and a high degree of coherence.

The MAX IV Laboratory currently operates two MX beamlines: BioMAX (Ursby *et al.*, 2020 ▸) and MicroMAX. Sirius (Liu *et al.*, 2014 ▸) and ESRF EBS (Raimondi, 2016 ▸) are other fourth-generation sources in operation with many others, in different stages of design and construction. The Swiss Light Source (SLS) at the PSI is scheduled to start the upgrade to SLS 2.0 in 2023 (Streun *et al.*, 2018 ▸).

The high degree of coherence is revolutionizing X-ray imaging (Thibault *et al.*, 2014 ▸) but the high brilliance is also opening up new possibilities in MX. By careful beamline design, the high brilliance of the source results in high brilliance at the sample allowing a high flux in a small beam focus of a highly parallel beam. At BioMAX, a photon flux of 10^13^ photons s^−1^ can be focused into a 20 × 5 µm full width at half-maximum (FWHM) spot with a divergence of 0.1 mrad. At MicroMAX, it will soon even be possible to focus >10^12^ photons s^−1^ (or >10^14^ photons s^−1^ with its multilayer monochromator) into a 1 × 1 µm spot with a divergence below 1 mrad, allowing higher time resolution and smaller samples to be measured.

### 1.2. JUNGFRAU detector

The extreme brilliance at XFELs triggered the renaissance of integrating detectors (Mozzanica *et al.*, 2012 ▸; Hart *et al.*, 2012 ▸; Hatsui & Graafsma, 2015 ▸). One of the major breakthroughs, allowing for practical use of the integrating technology, was the in-pixel adaptive gain. This technology enables the detector to operate with multiple dynamic ranges, which are dynamically switched from the highest gain to the lowest by individual pixels during exposure, allowing single-photon sensitivity for pixels with low incoming flux and high-dynamic range for pixels with high illumination. This development was essential for the megahertz pulse trains at the European XFEL and has been successfully introduced with the AGIPD detector (Henrich *et al.*, 2011 ▸; Allahgholi *et al.*, 2019 ▸). However, the low pixel depth limited its usability for slower applications, thus a new generation of integrating detectors, JUNGFRAU, was developed at the PSI (Mozzanica *et al.*, 2014 ▸).

JUNGFRAU was proven to be not only an excellent XFEL detector (Nass *et al.*, 2020 ▸) but also a promising system for synchrotron-based MX (Leonarski *et al.*, 2018 ▸), showing superiority over photon-counting systems at kilohertz frame rates. Yet, getting a detector excellent in acquiring kilohertz data is only the first step, as a new challenge is created – the data volume. A 4 MP sized detector, standard size for MX, with a 16-bit pixel counter depth, operating at a 2 kHz frame rate, produces a steady stream of 17 GB of data per second, which has to be handled by the downstream IT infrastructure, including data storage and analysis.

### 1.3. Jungfraujoch data-acquisition system

The JUNGFRAU data stream is a challenge for traditional CPU-only IT architecture (Leonarski *et al.*, 2020 ▸), since it would require a massive parallel readout system with multiple servers handling the incoming data. This not only leads to significant infrastructure and support cost but also calls for a sophisticated control and synchronization layer. To solve the issue, the PSI has therefore developed a control and readout system called Jungfraujoch, integrating a CPU, general-purpose graphical processing units (GPGPUs) and field-programmable gate array (FPGA) technologies, with a single server handling the full JUNGFRAU 4M 2 kHz data stream of 17 GB s^−1^ (Leonarski *et al.*, 2023 ▸). While developed at the PSI, we transferred and used the Jungfraujoch at MAX IV, which was relatively easy due to the compact design.

## 2. Materials and methods

### 2.1. Sample preparation

Lysozyme (Sigma–Aldrich) was dissolved in 100 m*M* sodium acetate, pH 3.0, to a final concentration of 25 mg ml^−1^. To obtain microcrystals, the lysozyme solution was mixed 1:1 with precipitant solution (22% NaCl, 6.4% PEG 6000 in 80 m*M* sodium acetate, pH 3.0) and incubated overnight. The resulting crystals had an average size distribution of 20 × 15 × 15 µm and were harvested by centrifugation. Cellulose matrix was prepared by dissolving 22%(*w*/*v*) 2-hydroxyethyl-cellulose in H_2_O, and left to swell overnight. For data collection, the crystals were embedded 1:4 in the cellulose matrix.

*Krokinobacter eikastus* rhodopsin 2 (KR2) samples and crystals were obtained as described before (Kato *et al.*, 2015 ▸; Skopintsev *et al.*, 2020 ▸). Briefly, the crystals were prepared by mixing buffered protein and monoolein in a 4:7(*v*/*v*) ratio. The formed lipidic cubic phase (LCP) was ejected into precipitant solution (200 m*M* sodium acetate, pH 4.4, 150 m*M* MgCl_2_, 34% PEG 200) and left to crystallize overnight in the dark at 20°C. The resulting crystals were plate-like, with dimensions of (20–35) × (20–35) × (1–3) µm. Before data collection, the crystal phase was washed in 150 m*M* NaCl, 34% PEG 200 solution, and mixed with fresh LCP prepared from monoolein and 1 *M* Tris, pH 9.0, 150 m*M* NaCl, 34% PEG 200 through a three-way syringe coupler (James *et al.*, 2019 ▸).

### 2.2. Beamline setup and data acquisition

**Figure 1:**
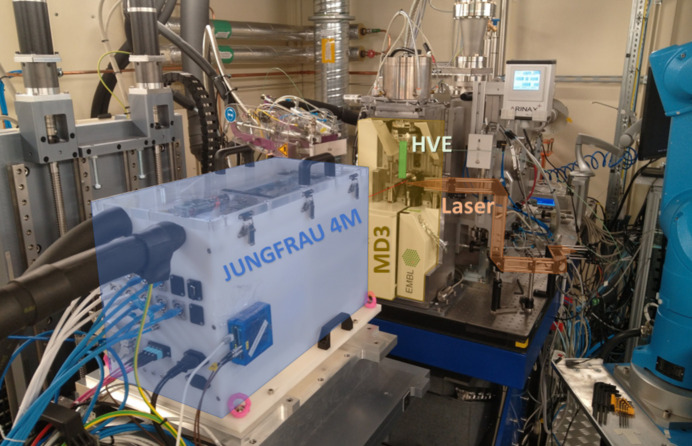
The setup at the BioMAX beamline. Blue represents the JUNGFRAU 4M prototype detector, yellow is the existing MD3 diffractometer, orange is the transient laser triggering setup and green is the mounted HVE.

The beamline setup at the BioMAX beamline was modified by replacing the standard EIGER 16M detector with the JUNGFRAU 4M detector, using the existing detector stage. The detector was integrated through the Jungfraujoch server (IBM IC922) to the MAX IV infrastructure (Fig. 1 ▸).

The HVE injector (Max Planck Institute for Medical Research, Heidelberg, Germany) used in this experiment was mounted vertically to the BioMAX micro-diffractometer (MD3, ARINAX, France). Protein crystals loaded into the HVE reservoir were extruded through tipped silica capillaries with an inner diameter of 75 µm by a high-performance liquid chromatography pump (Shimadzu, Japan, LC-20AD), and the sample jet was stabilized with helium as sheath gas (Shilova *et al.*, 2020 ▸). The X-ray beam was focused to a 20 × 5 µm FWHM spot at the sample position, and the beam energy was set to 11 and 15 keV at fluxes of 1.2 × 10^13^ and 6 × 10^12^ photons s^−1^, respectively, for optimal photon yield. At these values, a crystal receives a dose of less than 63 and 21 kGy, respectively, per millisecond exposure, well below the assumed room-temperature radiation limit of 300 kGy (Holton, 2009 ▸). For a comparison of different data-collection speeds, lysozyme microcrystals were extruded at speeds of 2.5 and 0.22 mm s^−1^ for data collection at 1 kHz and 100 Hz (0.1 kHz), respectively. The X-ray beam was attenuated for the 100 Hz acquisition by a factor of ten in order to have a similar dose per frame as at 1 kHz. For the time-resolved measurement of KR2 photo-dynamics, the sample was extruded at 1.5 mm s^−1^. As a pump trigger, a 530 nm laser diode (Roithner Lasertechnik GmbH, Austria) was mounted close to the sample area and focused onto the extruded sample to a 100 × 80 µm FWHM spot, offset with respect to the X-ray beam by ∼30°. Since the laser spot was larger than the X-ray spot, its position was slightly offset vertically so that the X-rays were co-aligned with the lower part of the laser. The fluence of the laser at the sample position was measured to be 12.7 W cm^−2^. The detector and laser were synchronized using two digital delay generators (DDGs) (DG645, Stanford Research Systems, USA). One DDG was used for generating pulses with a repetition rate of 7.5 Hz, defining the total probe length of 133.3 ms. This master clock was then connected to the second DDG, whose first output triggered the detector using a rising-edge transistor-to-transistor logic (TTL) pulse and, after a delay of 10 ms, whose second output triggered the pump laser for 10 ms using a rectangular pulse. This delay was added to compensate for any possible lag during triggering of the detector. The remaining 3.3 ms was a safety margin to ensure the system was ready for the next cycle. Correct timings were confirmed by measuring the TTL signals and the actual pump-laser output using a fast photo diode on an oscilloscope.

Sample centering and data acquisition were carried out with the beamline-control software *MXCuBE3* (Mueller *et al.*, 2017 ▸). Using the representational state transfer interface compatible with DECTRIS EIGER systems, the JUNGFRAU detector was integrated smoothly into the beamline-control system by adapting the existing EIGER control infrastructure. For the time-resolved experiments, pulse duration for the pump laser as well as the exposure time per image and number of images per trigger for the JUNGFRAU detector were easily configured via the *MXCuBE3* user interface.

### 2.3. JUNGFRAU and Jungfraujoch

The diffraction data were collected with the PSI-developed adaptive-gain charge-integrating JUNGFRAU detector (Leonarski *et al.*, 2018 ▸; Mozzanica *et al.*, 2018 ▸). This detector is composed of eight modules, comprising roughly 4 million pixels in total, with a single pixel size of 75 × 75 µm. The detector was operated at two different frame rates: (*a*) at 2 kHz, with 500 µs frame time and 480 µs integration time; and (*b*) at 1 kHz, with 1 ms frame time and 980 µs integration time. At these settings, the detector was streaming raw data at rates of 17 and 8 GB s^−1^, respectively (Leonarski *et al.*, 2020 ▸). Acquisition at 100 Hz was achieved by summing every ten frames with the detector operating at 1 kHz, similar to the intrinsic frame summation inside JUNGFRAU in standard operating mode.

Detector-gain calibration and pedestal-factor collection were performed using a procedure outlined previously (Redford *et al.*, 2018 ▸; Leonarski *et al.*, 2020 ▸), including pedestal-tracking correction to account for drift of the dark current. Dark images were collected before each measurement: a pedestal for high gain (G0) was calculated using 3000 dark frames collected at the same integration time and frame time as the actual measurement, while pedestals for medium gain (G1) and low gain (G2) were calculated based on 200 frames collected at the same integration time as the measurement, but with a reduced fixed frame rate of 100 Hz. To reduce the dark current, the detector was cooled to −10°C.

Detector control and data readout were performed with the Jungfraujoch server (Leonarski *et al.*, 2023 ▸). Here, network packets arriving from the detector are received by an FPGA board, which plays the role of a smart network-interface card. The FPGA board implements network-protocol decoding as well as conversion of JUNGFRAU raw frames to photon counts with pedestal and gain corrections. Converted images are written to CPU memory, and the CPU handles the assembly of full images, optional frame summation to reduce frame rate, and compression with the *Bitshuffle*/*LZ4* algorithm (Masui *et al.*, 2015 ▸). Additionally, assembled images are also sent, at a highly reduced rate, to the beamline consoles via a messaging queue, allowing the display of a live preview. Spot finding was implemented on the GPGPU, but the functionality was not mature enough during the beam time and was not used for live data processing.

### 2.4. Data processing and analysis

The *CrystFEL* 0.10 application suite (White *et al.*, 2012 ▸) was used for offline data analysis. Spot finding was performed using the *Peakfinder8* algorithm (Barty *et al.*, 2014 ▸) with spots of one or more pixels allowed, while signal-to-noise (SNR) and photon-counting thresholds were optimized separately for each crystal (Table 1 ▸). Indexing of the data was performed with the *XGANDALF* algorithm (Gevorkov *et al.*, 2019 ▸). Diffraction-geometry parameters, including beam center and detector distance, were iteratively optimized with detector-distance and geoptimiser tools included in the *CrystFEL* package. Scaling and post-processing were executed in *partialator* with the *xsphere* algorithm. Time-resolved data were saved with an additional hierarchical data format, version 5 (HDF5) virtual dataset, that pointed to images belonging to a particular time point. KR2 data were additionally treated with *STARANISO* (Tickle *et al.*, 2016 ▸) to account for anisotropic diffraction. Difference maps were calculated using *Phenix* (Liebschner *et al.*, 2019 ▸) and figures were generated with *PyMOL* (Schrödinger, LLC, 2015 ▸).

## 3. Results and discussion

### 3.1. Detector integration

**Figure 2:**
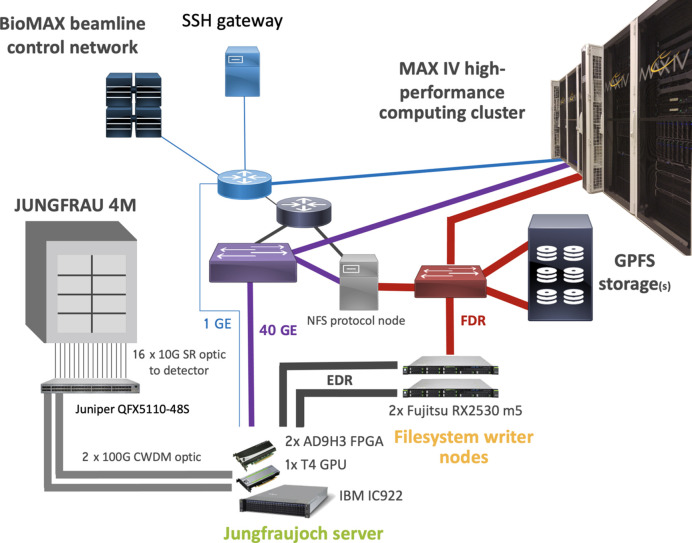
Components and connection of the Jungfraujoch data-acquisition system used at MAX IV. The following networks were used for the experiment: (gray/black) a network specifically installed for the experiment, (red) InfiniBand fabric for file-system access, (purple) a fast (40 Gbit s^−1^) Ethernet network for streaming and (blue) a slow (1 Gbit s^−1^) control network.

The 17 GB s^−1^ stream of data from the JUNGFRAU detector was served to a single edge server for data acquisition and preliminary analysis. The server was an IBM IC922 system, consisting of two POWER9 CPUs, two Alpha Data 9H3 boards with Xilinx Virtex Ultrascale+ high bandwidth memory (HBM) FPGAs running the Jungfraujoch firmware (Leonarski *et al.*, 2023 ▸), a single Nvidia T4 GPGPU and two Mellanox Connect-X 5 Ex InfiniBand host channel adapters. The connection between the beamline switch and the two server FPGAs was patched with a long-range, 100 Gbit s^−1^, optical connection. The detector modules, the detector switch and the receiving FPGAs formed a dedicated data network between the beamline network and the MAX IV server hall using 2 out of 16 existing fiber optic cables. Hosting the edge server in the central MAX IV server room allows a short-distance InfiniBand enhanced data rate (EDR) (100 Gbit s^−1^) network connection to the standard MAX IV x86_64 computing infrastructure (Fig. 2 ▸). This allows data processed and compressed with the Jungfraujoch system to be streamed to a single x86_64 server hosting the Jungfraujoch file-writer application, writing data following the NXmx gold standard (Bernstein *et al.*, 2020 ▸) to the central MAX IV GPFS storage. The bandwidth of the compressed data to the MAX IV storage was only limited by the server-to-storage network connection (FDR InfiniBand, 56 Gbit s^−1^) and the capabilities of the HDF5 file writer. With a performance test, we established that a simple single-threaded HDF5 writer can reach a throughput of higher than 4 GB s^−1^ with the MAX IV infrastructure. This allowed for continuous data acquisition at 2 kHz, with a compression rate slightly above 4. The MAX IV edge cloud infrastructure is being upgraded to 100 Gbit s^−1^ Ethernet at the time of writing of this article. Together with ongoing development of the Jungfraujoch system, this will allow the file writing of a compressed JUNGFRAU detector data stream on standard MAX IV data-acquisition nodes.

### 3.2. Faster SSX data acquisition

To investigate if an increased frame rate could have a detrimental effect on data quality, we compared lysozyme data acquired at 1 kHz (PDB ID 8p1c) with data acquired at a more ‘standard’ 100 Hz rate (PDB ID 8p1d). For the purpose of the comparison, we adjusted the X-ray beam transmission and jet speed, so that both X-ray dose and illuminated sample area were comparable. As summarized in Table 1 ▸, resolution, indexing rate, CC_1/2_ and SNR were comparable for both data-collection modes. This result is in line with the previous comparison on rotational crystallography data quality with the JUNGFRAU detector, which demonstrated that increasing data-collection speed with higher photon flux is not detrimental to protein-crystal data quality (Leonarski *et al.*, 2018 ▸). Though the kilohertz data collection shows a minor advantage in the statistics, we believe that the difference is not significant, as some experimental parameters, for example jet speed, cannot scale exactly by a factor of ten.

### 3.3. Kilohertz continuous data acquisition

Next, to assess the full capabilities of the detector and data-acquisition system, we collected diffraction data of lysozyme crystals at 2 kHz frame rate. In the first experiment, we collected raw data without any compression, expecting that the resulting data rate of 17 GB s^−1^ is beyond the capability of the network and storage infrastructure. In this mode, we were able to collect roughly 22 000 frames before running out of intermediate storage space, which marks the burst capability of the system. Subsequently, we enabled the conversion and compression mode. To ensure this mode allows for continuous measurement, we aimed to collect 500 000 images, which is an order of magnitude higher than the burst capability. The resulting 500 000 image lysozyme dataset was collected in 4 min and 10 s, without any lost frames (PDB ID 8p1b). The compression factor of the data was roughly sevenfold, resulting in a proven decrease in detector data rate from 17 GB s^−1^ in raw mode to a 2.4 GB s^−1^ compressed data rate. To evaluate the quality of data acquired in a short time, a subset of the data of 10 000 images, collected in 5 s, was randomly selected (frames 40 001–50 000). This subset was processed using the same parameters as the full dataset and a 2.05 Å resolution structure could be obtained (PDB ID 8p1a). A summary of the datasets is found in Table 1 ▸ and the raw data are accessible at https://doi.org/10.48391/b0c36bb8-a00c-4519-8dcc-08d5ca60a313.

### 3.4. Kilohertz time-resolved data acquisition

In marine bacteria, light-driven sodium pumps maintain a low intracellular sodium-ion concentration and membrane potential (Inoue *et al.*, 2013 ▸). These proteins are members of the light sensing and harvesting rhodopsin family (Govorunova *et al.*, 2017 ▸; Terakita, 2005 ▸), of which KR2 is a prototypical member. Its mechanism has been studied extensively by time-resolved spectroscopy (Inoue *et al.*, 2013 ▸; Tahara *et al.*, 2015 ▸, 2018 ▸; Hontani *et al.*, 2016 ▸; Kaur *et al.*, 2019 ▸; Chen *et al.*, 2018 ▸; Asido *et al.*, 2019 ▸), as well as X-ray crystallography (Kato *et al.*, 2015 ▸; Gushchin *et al.*, 2015 ▸; Kovalev *et al.*, 2019 ▸; Skopintsev *et al.*, 2020 ▸), making it an ideal model system to demonstrate the kilohertz time-resolved data acquisition in this work.

**Figure 3:**
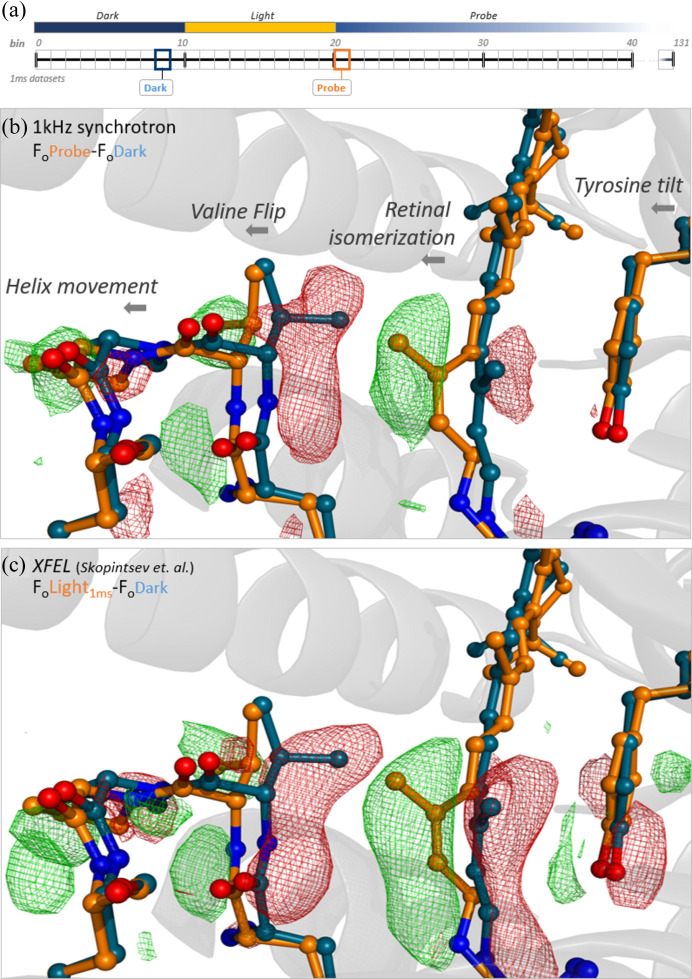
(*a*) A schematic explanation of the data-collection pattern used to record the millisecond datasets. (*b*) A difference map of *F*_o_ from bin #21 (probe, 1 ms after illumination) minus *F*_o_ of bin #8 (dark). (*c*) A difference map of 1 ms *F*_o_ (PDB ID 6tk2) minus dark *F*_o_ (PDB ID 6tk6) recorded at SwissFEL [data from Skopintsev *et al.* (2020 ▸)]. All the maps are shown at ±3σ and resolution was cut at 2.38 Å.

Using the pump–probe setup described, we collected ∼29 000 diffraction patterns for each of the one-millisecond-resolution time points, covering the range 1–130 ms. This time range was chosen deliberately, as we needed to ensure that exposed crystals were completely clearing the interaction area between pump–probe events. With the sample jet speed at 1.5 mm s^−1^, the average crystal travels 200 µm in 130 ms, ensuring clearance of the 100 µm diameter laser spot. The pump laser was turned on 10 ms after the detector trigger to ensure complete initialization. The laser was on for 10 ms, and the crystal diffraction was probed for an additional 110 ms after the pump event. At a 10% indexing rate, the complete dataset, comprising 120 time points with 29 000 indexed frames each, was collected in roughly 10 h, allowing for a very efficient data collection [Fig. 3 ▸(*a*)].

Comparison of the dark structure obtained here with those previously measured (Skopintsev *et al.*, 2020 ▸) showed no significant difference at the achieved resolution of ∼2.3 Å. The example in Fig. 3 ▸(*b*) uses the time bin #21, which corresponds to 10–11 ms after laser illumination was initiated and 0–1 ms after illumination ended. The *F*_o_probe–*F*_o_dark difference map [Fig. 3 ▸(*b*)] clearly shows the retinal isomerization, the valine flip and the movement of the α-helix, which are characteristic features occurring in the millisecond time range of the KR2 photocycle, as observed previously (Skopintsev *et al.*, 2020 ▸).

The comparison shows that the 1 kHz synchrotron difference map is comparable to the 1 ms SwissFEL result (Skopintsev *et al.*, 2020 ▸) [Fig. 3 ▸(*c*)]. However, because of limitations in dose and due to radiation damage when collecting synchrotron data at room temperature, the XFEL dataset benefits from a more powerful X-ray beam and therefore contains information at higher resolution. The synchrotron map shows less defined densities than the XFEL map, which allows one to resolve small amino acid movements more precisely, as can be seen around tyrosine 218 [Figs. 3 ▸(*b*) and 3 ▸(*c*)]. However, most of the features observed at 1 ms are present in both maps. This shows that kilohertz data acquisition at a fourth-generation synchrotron, combined with a fast detector like the JUNGFRAU, is perfectly suitable for time-resolved studies on dynamics in the millisecond time range and possibly even below.

## 4. Raw data

The presented lysozyme structures for 100 Hz, 1 kHz, 2 kHz and 2 kHz subset are available in the Protein Data Bank (PDB) under the PDB accession codes 8p1d, 8p1c, 8p1b and 8p1a, respectively. Additionally, the raw diffraction datasets for lysozyme measured at 100 Hz, 1 kHz and 2 kHz are available for download at https://doi.org/10.48391/b0c36bb8-a00c-4519-8dcc-08d5ca60a313.

## 5. Conclusions and outlook

With the advent of a new era of protein crystallography, focusing on more dynamic and biologically relevant experiments, accessibility to beam time and methodology has become a bottleneck. The measurement times at XFELs are very limited, so enabling the use of the more readily available synchrotron beam time is crucial. For this to work, the most brilliant fourth-generation sources are needed and measurement time needs to be utilized as efficiently as possible. Collecting time-resolved data as fast as possible at the available X-ray sources is a major step towards infusing the field by effectively providing more experiment time.

Here, we demonstrated the ability of the Jungfraujoch setup to collect a whole serial crystallography dataset with 5000 indexed frames in less than 5 s, without any significant loss in data quality. Furthermore, we showed that it is possible to push the time resolution for synchrotron-based time-resolved experiments to the low millisecond regime, all the while collecting multiple time points simultaneously, making these beam times highly efficient. With improvements on the detector side already pointing towards even faster speeds, and in combination with the advent of more fourth-generation sources and the Jungfraujoch data-acquisition architecture, it is thinkable to get into the microsecond domain in the near future, closing the gap to the ultra-fast XFEL-based measurements. The first beamline to push this frontier will be the upcoming MicroMAX beamline, which is tailored towards these kinds of experiments. Also, after the upcoming upgrade to SLS 2.0, the new PXI-VESPA beamline will focus on serial crystallography experiments, with its combination of new detectors, X-ray chopper and optional pink X-ray beam, targeting the 10 kHz data rate. Acquiring data at multiple kilohertz frame rates places a significant challenge on data infrastructure, but we were able to show that these challenges can be overcome. Since the kilohertz data are not noticeably worse compared with lower acquisition rates, it is suggested to always collect as fast as possible – here, faster is better. 

## Supplementary Material

PDB reference: lysozyme subset collected at 2 kHz in 5 s, 8p1a

PDB reference: lysozyme collected at 2 kHz, 8p1b

PDB reference: lysozyme collected at 1 kHz, 8p1c

PDB reference: lysozyme collected at 100 Hz, 8p1d

## Figures and Tables

**Table 1:** Data collection statistics and parameters used for spot finding Values in parentheses refer to the highest resolution bin.

	Lysozyme	Lysozyme	Lysozyme	Lysozyme	KR2	KR2
	1 kHz	0.1 kHz	2 kHz subset	2 kHz	Dark	Probe
Frame rate (Hz)	1000	100	2000	2000	1000	1000
Collection time (s)	100	1000	5	250	270	270
Photon energy (keV)	15	15	11	11	11	11
Photon flux (photons s^−1^)	6.0 × 10^12^	6.0 × 10^11^	1.2 × 10^13^	1.2 × 10^13^	1.2 × 10^13^	1.2 × 10^13^
Beam transmission (%)	100	10	100	100	100	100
Jet speed (mm s^−1^)	2.5	0.22	4.9	4.9	1.5	1.5
Number of frames	100000	100000	10000	500000	269302	269302
Number of indexed crystals	93717	71623	5412	265411	29341	28812
Resolution (Å)	55.90–1.58 (1.64–1.58)	55.90–1.60 (1.66–1.60)	39.15–2.05 (2.12–2.05)	39.15–1.70 (1.76–1.70)	58.89–2.30 (2.38–2.30)	58.89–2.30 (2.38–2.30)
Completeness (%)	100	100	99.9	100	100	100
CC_1/2_	99.6 (56.6)	99.8 (47.4)	91.5 (52.2)	99.8 (54.3)	97.0 (47.3)	96.5 (42.1)
SNR ratio	9.3 (0.7)	10.6 (1.1)	5.4 (1.7)	16.3 (0.04)	5.6 (0.25)	5.5 (0.2)
Spot-finding parameters						
Threshold (–threshold =)	10	10	10	10	16	16
SNR ratio (–min-snr =)	3	4	3	3	6	6
Minimum pixel count (–min-pix-count =)	1	1	1	1	1	1
